# Improvement in Protein-Losing Gastroenteropathy Due to Gastric Polyposis by Laparoscopic Total Gastrectomy: A Case Report

**DOI:** 10.70352/scrj.cr.25-0407

**Published:** 2025-10-17

**Authors:** Manatsu Mizuno, Takuro Saito, Shigeto Nakai, Takaomi Hagi, Kota Momose, Kotaro Yamashita, Koji Tanaka, Tomoki Makino, Tsuyoshi Takahashi, Yukinori Kurokawa, Hidetoshi Eguchi, Yuichiro Doki

**Affiliations:** 1 Department of Gastroenterological Surgery, The University of Osaka Graduate School of Medicine, Suita, Osaka, Japan; 2 Department of Clinical Research in Tumor Immunology, The University of Osaka Graduate School of Medicine, Suita, Osaka, Japan

**Keywords:** juvenile polyposis syndrome, laparoscopic total gastrectomy, protein-losing gastroenteropathy

## Abstract

**INTRODUCTION:**

Juvenile polyposis syndrome (JPS) is a rare disease characterized by multiple hamartomatous polyps in the gastrointestinal tract that may cause protein-losing gastroenteropathy. In such cases, symptoms such as hypoalbuminemia and anemia are often difficult to manage. Although clinical guidelines provide recommendations for diagnosis and management, standardized treatment strategies remain to be fully established.

**CASE PRESENTATION:**

A 40-year-old man underwent laparoscopic total gastrectomy for protein-losing gastroenteropathy secondary to gastric polyposis. He had a history of colorectal polyposis associated with an *SMAD4* mutation and had previously undergone subtotal colectomy. The patient developed worsening anemia and hypoproteinemia. Upper gastrointestinal endoscopy revealed an increase in both the size and number of gastric polyps. Protein-losing gastroenteropathy was diagnosed using technetium-99m human serum albumin scintigraphy. Laparoscopic total gastrectomy with Roux-en-Y reconstruction was performed to control hypoproteinemia and eliminate the risk of malignant transformation of the polyps. Postoperatively, the symptoms resolved, and oral intake improved. He remained in good health and has continued a normal daily life without symptom recurrence for 4 years postoperatively.

**CONCLUSIONS:**

We present a case of JPS with an *SMAD4* mutation causing protein-losing gastroenteropathy and refractory anemia, successfully treated with laparoscopic total gastrectomy. The patient’s sustained nutritional status suggests that total gastrectomy may be an effective treatment option for gastric JPS with protein-losing gastroenteropathy.

## Abbreviations

99mTc-HSA
: technetium-99m human serum albumin
JPS
: juvenile polyposis syndrome

## INTRODUCTION

Juvenile polyposis syndrome (JPS) is a rare disorder characterized by multiple hamartomatous polyps in the gastrointestinal tract. It is an autosomal dominant hereditary disease, with pathogenic variants in the tumor suppressor genes, *SMAD4* or *BMPR1A*, identified in approximately 60% of cases.^1,2)^ Polyps most frequently occur in the colon (98%), followed by the stomach (14%).^3)^ In cases of gastric JPS, symptoms such as anemia due to bleeding, hypoalbuminemia, hypoproteinemia, and edema caused by protein-losing enteropathy are commonly observed. Several reports have described symptomatic improvement with surgical intervention.^4)^ Furthermore, patients with gastric JPS harboring *SMAD4* mutations have a 30% risk of developing gastric cancer.^5,6)^

Herein, we report a case of gastric polyposis with protein-losing gastroenteropathy that was refractory to medical treatment. Given the risk of malignant transformation, laparoscopic total gastrectomy was performed. The patient maintained a stable nutritional status and serum protein levels for 4 years postoperatively.

## CASE PRESENTATION

**Fig. 1:**
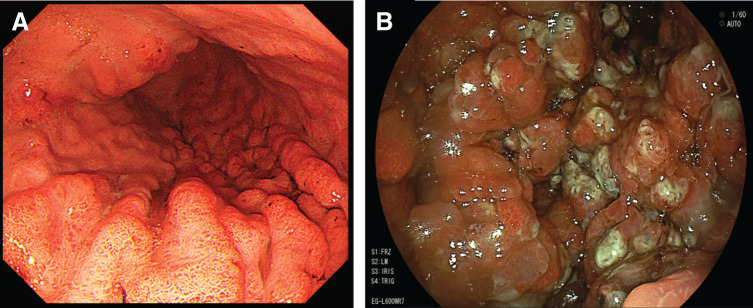
Endoscopic findings. (**A**) Three years prior to admission, no polyps were observed in the gastric body. (**B**) At the time of admission, a marked increase in both the size and number of polyps in the gastric body was observed.

**Fig. 2:**
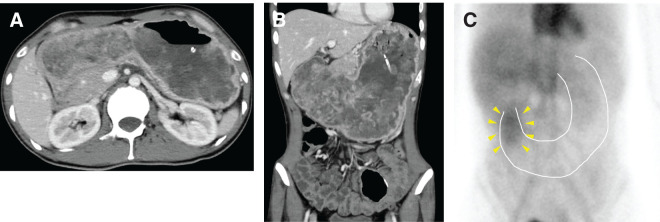
CT findings in axial (**A**) and coronal (**B**) views. The stomach is significantly enlarged, and numerous polyps are observed inside. (**C**) Technetium-99m human serum albumin scintigraphy demonstrated increased tracer accumulation in the gastric antrum 6 h after injection. White dotted lines: gastric outline; yellow arrowheads, tracer accumulation.

A 40-year-old male visited our hospital with a 2-week history of persistent vomiting and was admitted urgently. Twenty years ago, he underwent subtotal colectomy for isolated JPS due to an *SMAD4* mutation and had been under regular follow-up in the Department of Gastroenterology. Three years before admission, he experienced melena, followed by progressive anemia and hypoalbuminemia. Upper gastrointestinal endoscopy revealed a polyp surrounding the gastric cardia; however, biopsy showed no evidence of malignancy. During annual surveillance endoscopies, no significant changes were observed, and no polyps were detected in the gastric body (**Fig. 1A**); however, at the time of the current hospitalization, endoscopy revealed markedly enlarged polyps (**Fig. 1B**). Capsule endoscopy was also performed as a screening examination for small intestinal lesions 1 year ago, which revealed no apparent polyps in the small intestine. Blood tests at admission showed anemia (hemoglobin: 10.8 g/dL) and hypoalbuminemia (albumin: 2.2 g/dL), while tumor marker levels were within normal limits. Contrast-enhanced CT demonstrated multiple gastric polyps and significant gastric dilation (**Fig. 2A** and **2B**).

Initial management involved gastric decompression using a nasogastric tube, but drainage remained high (1.5–2.0 L/day), and the patient continued to experience persistent diarrhea (15–20 episodes per day). Technetium-99m human serum albumin (99mTc-HSA) scintigraphy demonstrated increased tracer accumulation in the antrum 6 h after injection (**Fig. 2C**), indicating protein-losing gastroenteropathy originating from the gastric antrum due to juvenile polyposis. Blood transfusions and albumin infusion were administered, but these symptoms proved refractory to treatment. Therefore, surgical resection was deemed necessary due to refractory anemia, hypoalbuminemia, gastric outlet obstruction, and the potential risk of malignant transformation.

**Fig. 3:**
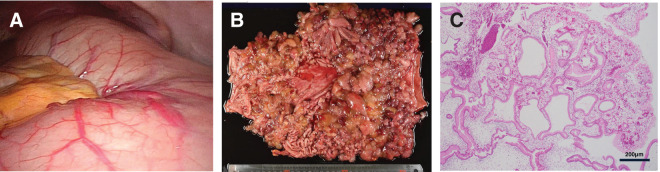
Surgical and pathological findings. (**A**) Intraoperative findings revealed a markedly distended stomach with limited visualization. (**B**) Multiple polyps were observed throughout the gastric mucosa in the resected specimen. (**C**) Histopathological examination revealed hyperplasia of the glandular epithelium accompanied by stromal edema. No evidence of malignancy was identified in any of the polyps or lymph nodes.

Laparoscopic total gastrectomy with Roux-en-Y reconstruction was subsequently performed, with an operative time of 229 min and blood loss of 100 mL. Intraoperative findings revealed a markedly distended stomach with limited visualization, which made standard laparoscopic port placement and exploration mildly difficult (**Fig. 3A**). Nevertheless, the procedure was completed without complications. Postoperatively, an umbilical surgical site infection was observed, which was likely related to preexisting hypoproteinemia. Diarrhea improved to 5–10 times per day, and oral intake was successfully resumed. The patient was discharged 26 days postoperatively.

Histopathological examination revealed foveolar epithelial hyperplasia and stromal edema (**Fig. 3B** and **3C**). No evidence of malignancy was identified in any of the polyps or resected lymph nodes.

**Fig. 4:**
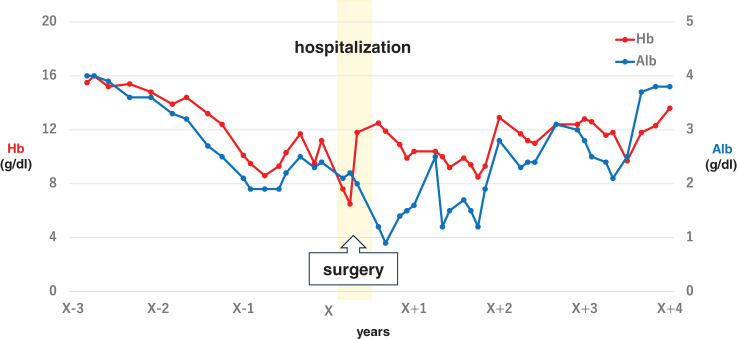
Pre- and postoperative changes in serum Alb and Hb levels. Alb, albumin; HB, hemoglobin

Following surgery, both anemia and hypoproteinemia gradually improved, and the patient was able to maintain adequate oral intake. He remains in good health and has continued a normal daily life without symptom recurrence for 4 years postoperatively (**Fig. 4**).

## DISCUSSION

JPS is characterized by hamartomatous polyps in the gastrointestinal tract. Approximately 75% of cases exhibit an autosomal dominant inheritance pattern.^7)^ As most JPS cases are caused by genetic mutations, no curative pharmacological treatments have been established even in the clinical guidelines, as the effectiveness of molecular targeted therapies or prophylactic medications has not been demonstrated.^8)^ Endoscopic polypectomy is recommended for symptomatic lesions, while surgical resection is indicated in cases with invasive carcinoma, intussusception, or when anemia and hypoproteinemia are difficult to control.^3)^ Pharmacological management is generally supportive and symptomatic, including iron supplementation or blood transfusions for anemia, albumin replacement for hypoalbuminemia, and oral nutritional supplements or total parenteral nutrition for malnutrition. In the present case, gastric decompression using a nasogastric tube, blood transfusions, and albumin infusion were performed as part of the preoperative management. In addition, JPS is associated with malignant potential, and in patients with gastric involvement harboring *SMAD4* mutations, the risk of developing gastric cancer has been estimated to be as high as 30%.^5,6)^ Therefore, surgical intervention should be considered at the time of diagnosis in appropriate cases. Given the increased risk of postoperative complications, including anastomotic leakage, owing to the poor general condition associated with concurrent anemia and hypoproteinemia, careful perioperative management is essential to ensure patient safety.

We encountered a case of JPS with an *SMAD4* mutation that presented with protein-losing gastroenteropathy. As the anemia and hypoproteinemia were refractory to medical treatment and the gastric polyps had increased in size, surgical intervention was considered necessary because of the associated risk of malignant transformation. Surgical options for gastric JPS include both total gastrectomy and partial gastrectomy, depending on the distribution of polyps. In our case, numerous polyps were widely distributed from the cardia to the gastric body, necessitating total gastrectomy. In surgical approaches for gastric JPS, obtaining an adequate surgical field is often challenging because of the difficulty in decompressing a stomach distended with numerous polyps.^9)^ A review of 30 reported cases in Ichushi (Igaku Chuo Zasshi: a Japanese medical literature database) of patients who underwent total gastrectomy for JPS revealed that 13 involved laparoscopic surgery using a 5-port technique^4,9–36)^ (**Table 1**). Laparoscopic surgery is considered to facilitate access to the entire abdominal cavity with minimal disruption of the abdominal wall compared to open surgery. In cases such as JPS, where the stomach is highly dilated, the advantages of laparoscopic surgery may be particularly significant. In the present case, although the procedure was performed laparoscopically, the stomach was markedly distended and edematous, making visualization challenging. However, by carefully manipulating the thickened gastric wall to secure the operative field, surgery was completed safely without injury or significant bleeding. No established guidelines currently exist for lymph node dissection in cases where gastric cancer is not confirmed preoperatively. However, considering the potential for carcinoma on histopathological examination, D1 lymphadenectomy was performed.

**Table 1:** Thirty cases of total gastrectomy for juvenile gastric polyposis in Japan

No.	Author	Year	Sex	Age	Polyp location	Anemia/low protein	99mTc-HAS scintigraphy	Cancer	Cancer location	Lap/open	Number of ports	Improvement in anemia/low protein	Postoperative follow-up period (months)	Treatment for colorectal polyposis
1	Hamamoto	1994	F	36	Fundus–body	−/−	–	+	Fundus	Open		nd/nd	nd	–
2	Hirata	2000	F	63	Whole	+/+	–	–		Open		nd/nd	nd	–
3	Furukawa	2000	M	57	Whole	+/+	–	+	Antrum	Open		nd/nd	nd	–
4	Harada	2001	M	49	Whole	+/+	–	–		Open		+/+	nd	–
5	Komatsu	2004	M	60	Whole	+/+	–	–		Open		+/+	6	–
6	Yamaguchi	2004	M	48	Whole	+/+	–	–		Open		+/+	24	–
7	Yamanaka	2008	M	44	Upper–lower body	−/−	–	+	Middle body	Open		nd/nd	12	–
8	Yagi	2009	F	60	Whole	+/+	–	+	Antrum	Open		+/+	nd	–
9	Takashima	2009	F	50	Middle body–antrum	−/+	–	+	Antrum	Open		+/+	30	–
10	Yamashita	2009	M	28	Whole	+/+	+	–		Open		+/+	nd	Colorectal polypectomy
11	Ozawa	2010	F	48	Whole	+/+	–	+	Antrum	Open		nd/nd	24	–
12	Matsui	2010	M	39	Whole	+/+	–	–		Open		+/+	nd	–
13	Mizuuchi	2011	F	47	Upper body–antrum	+/+	–	–		Lap	5 ports	+/+	2	–
14	Okubo	2013	F	42	Whole	+/+	–	–		Open		+/+	4	–
15	Nagasue	2013	F	42	Whole	+/+	–	–		Lap	nd	+/+	nd	–
16	Honda	2013	F	29	Body–antrum	+/+	–	–		Lap	nd	nd/nd	nd	No treatment
17	Sato	2014	F	28	Middle body–pylorus	+/+	–	–		Lap	5 ports	+/+	1	–
18	Suzuki	2015	F	49	Whole	+/+	–	–		Lap	5 ports	+/+	12	–
19	Matsuo	2015	F	37	Whole	+/+	–	+	Antrum	Lap	nd	nd/nd	nd	–
20	Ochi	2016	F	50	Whole	+/+	–	+	Antrum	Open		nd/nd	nd	–
21	Yube	2018	F	48	Whole	+/+	–	+	Whole	Lap	nd	nd/nd	7	–
22	Jogo	2018	M	63	Whole	+/+	–	+	Lower body	Lap	nd	nd/nd	16	–
23	Nakagawa	2019	M	48	Whole	+/+	–	–		Open		+/+	9	–
24	Ito	2022	F	43	Whole	+/+	–	+	Antrum	Lap	nd	nd/nd	nd	–
25	Sakai	2022	F	57	Whole	+/+	–	+	Middle body	Lap	5 ports	nd/nd	12	–
26	Takahashi	2023	F	53	Whole	+/+	–	–		Lap	5 ports	nd/nd	nd	–
27	Niya	2023	F	42	Whole	+/+	–	+	Antrum	Open		nd/nd	14	–
28	Utsunomiya	2023	F	70	Upper–middle body	+/+	–	+	Body	Lap	nd	+/+	nd	–
29	Nakamura	2024	M	33	Stomach–duodenum	+/+	–	–		Open (TG + PD)		nd/nd	18	–
30	Our case	–	M	40	Whole	+/+	+	–		Lap	5 ports	+/+	48	Subtotal colectomy

99mTc-HSA, technetium-99m human serum albumin; F, female; M, male; nd, not described; PD, pancreaticoduodenectomy; TG, total gastrectomy

Several reports have described improvements in anemia and hypoproteinemia following surgical intervention, as well as favorable recurrence-free postoperative outcomes in cases of gastric cancer associated with JPS. Previous reports of total gastrectomy for JPS showed that the course of the symptoms was described in 13 among 30 patients, all of whom experienced improvement in postoperative anemia and hypoalbuminemia. However, the follow-up periods ranged from 2 to 24 months, which is shorter than in our patient who had a 48-month follow-up^4,9–36)^ (**Table 1**). Moreover, previous studies have reported that approximately 27.4% of JPS cases exhibit polyps in both the stomach and intestine.^37)^ Although there are documented cases in which patients underwent gastrectomy with colorectal polypectomy^4)^ or colectomy with gastric endoscopic mucosal resection,^38)^ there have been no reports in Japan of patients undergoing both gastrectomy and colectomy. Regarding gastric cancer, there are case reports documenting recurrence-free survival at 15 months postoperatively.^13)^ In the present case, the patient was followed up for 4 years postoperatively, maintaining stable hemoglobin and serum albumin levels, without symptom exacerbation and with adequate oral intake. Our report represents the longest follow-up demonstrating sustained symptomatic and biochemical improvement among reported cases in Japan. Given that JPS patients remain at risk of developing new polyps and potential malignancies in other parts of the gastrointestinal tract over time, long-term surveillance is therefore essential to monitor for recurrence and to manage nutritional complications such as anemia and hypoalbuminemia, thereby ensuring timely intervention and improved patient outcomes.

## CONCLUSIONS

We encountered a case of JPS with an *SMAD4* mutation who presented with protein-losing gastroenteropathy and medically refractory anemia and hypoproteinemia, for which laparoscopic total gastrectomy was performed. The patient maintained good nutritional status throughout the long-term postoperative course. Therefore, total gastrectomy may be an effective therapeutic option for symptom control in patients with gastric JPS associated with protein-losing gastroenteropathy.

## DECLARATIONS

### Availability of data and materials

The data that support the findings of this study are available upon request from the corresponding author, Takuro Saito. The data are not publicly available as they contain information that could compromise the privacy of research participants.
